# Searching for improvements in predicting human eye colour from DNA

**DOI:** 10.1007/s00414-021-02645-5

**Published:** 2021-07-14

**Authors:** Magdalena Kukla-Bartoszek, Paweł Teisseyre, Ewelina Pośpiech, Joanna Karłowska-Pik, Piotr Zieliński, Anna Woźniak, Michał Boroń, Michał Dąbrowski, Magdalena Zubańska, Agata Jarosz, Rafał Płoski, Tomasz Grzybowski, Magdalena Spólnicka, Jan Mielniczuk, Wojciech Branicki

**Affiliations:** 1 Faculty of Biochemistry, Biophysics and Biotechnology, Jagiellonian University, Kraków, Poland; 2 Malopolska Centre of Biotechnology of the Jagiellonian University, Kraków, Poland; 3 Institute of Computer Science, Polish Academy of Sciences, Warsaw, Poland; 4 Faculty of Mathematics and Information Science, Warsaw University of Technology, Warsaw, Poland; 5 Faculty of Mathematics and Computer Science, Nicolaus Copernicus University in Toruń, Toruń, Poland; 6 Institute of Environmental Sciences, Faculty of Biology, Jagiellonian University, Kraków, Poland; 7 Central Forensic Laboratory of the Police, Warsaw, Poland; 8 Laboratory of Bioinformatics, Neurobiology Centre, Nencki Institute of Experimental Biology of Polish Academy of Sciences, Warsaw, Poland; 9 Faculty of Law and Administration, Department of Criminology and Forensic Sciences, University of Warmia and Mazury in Olsztyn, Olsztyn, Poland; 10 Unit of Forensic Sciences, Faculty of Internal Security, Police Academy, Szczytno, Poland; 11 Department of Medical Genetics, Warsaw Medical University, Warsaw, Poland; 12 Division of Molecular and Forensic Genetics, Department of Forensic Medicine, Nicolaus Copernicus University in Toruń, Collegium Medicum in Bydgoszcz, Bydgoszcz, Poland

**Keywords:** Whole-exome sequencing, Eye colour, DNA phenotyping, Predictive modelling, Machine learning algorithms

## Abstract

Increasing understanding of human genome variability allows for better use of the predictive potential of DNA. An obvious direct application is the prediction of the physical phenotypes. Significant success has been achieved, especially in predicting pigmentation characteristics, but the inference of some phenotypes is still challenging. In search of further improvements in predicting human eye colour, we conducted whole-exome (enriched in regulome) sequencing of 150 Polish samples to discover new markers. For this, we adopted quantitative characterization of eye colour phenotypes using high-resolution photographic images of the iris in combination with DIAT software analysis. An independent set of 849 samples was used for subsequent predictive modelling. Newly identified candidates and 114 additional literature-based selected SNPs, previously associated with pigmentation, and advanced machine learning algorithms were used. Whole-exome sequencing analysis found 27 previously unreported candidate SNP markers for eye colour. The highest overall prediction accuracies were achieved with LASSO-regularized and BIC-based selected regression models. A new candidate variant, rs2253104, located in the *ARFIP2* gene and identified with the HyperLasso method, revealed predictive potential and was included in the best-performing regression models. Advanced machine learning approaches showed a significant increase in sensitivity of intermediate eye colour prediction (up to 39%) compared to 0% obtained for the original IrisPlex model. We identified a new potential predictor of eye colour and evaluated several widely used advanced machine learning algorithms in predictive analysis of this trait. Our results provide useful hints for developing future predictive models for eye colour in forensic and anthropological studies.

**Supplementary Information:**

The online version contains supplementary material available at 10.1007/s00414-021-02645-5.

## Introduction

Increasing understanding of human genome variability is enabling better use of DNA’s predictive potential [1]. Besides clinical applications, predictive DNA analysis can be useful in forensics for intelligence purposes [2], in molecular anthropology [3] and in identification of historical figures [4–6]. In recent years, intensive research has been carried out on the prediction of various human appearance characteristics [e.g. 7–15]. The most significant progress was made in the prediction of pigmentation characteristics, and eye colour in particular [16]. Nevertheless, the genetic architecture of some categories of pigmentation phenotypes remains elusive, their prediction is still inaccurate and research to improve accuracy continues. One such category is intermediate eye colour, which in the most commonly used IrisPlex model is predicted with low sensitivity [16]. Because of the very complex genetic basis of the appearance traits, a promising direction is building predictive tools that take into account markers based on the criterion of improved prediction and not genetic association, and the use of more advanced mathematical methods in prediction modelling [17]. There are many machine learning (ML) methods available for developing predictive models, and their effectiveness may depend on the type and amount of data used; some of them may be more suitable than others for taking into account diverse genetic phenomena, including epistasis. First, we can distinguish linear and nonlinear methods [18]. The linear methods in their basic form are limited to detecting the linear dependency between a class variable and attributes. Representative examples are logistic and multinomial regression, linear discriminant analysis (LDA), the basic linear version of support vector machines (SVM) or perceptron. The nonlinear methods are designed to detect more complex dependencies between a class variable and attributes. Examples include various tree-based methods, multivariate adaptive regression splines (MARS) and multilayer neural networks (NN). The advantage of the first group is the relatively low computational cost of fitting the model as well as simplicity and interpretability. On the other hand, nonlinear models usually achieve greater predictive power, especially in the case of complex classification issues. Moreover, they are also able to detect interactions among attributes [19]. In addition to single models, ensemble techniques, which combine multiple learning algorithms, have gained great popularity. It has been proved that ensemble methods such as random forest (RF) or extreme gradient boosting (XGB) are among the most powerful classification models; they usually achieve significantly higher accuracy when compared to simple models. The price for this is the higher computational cost and more complicated interpretation. An important line of research in ML is focused on combining classification methods with feature selection techniques. Feature selection plays a crucial role in many analyses, especially when the number of attributes is large compared with the sample size. Selection of relevant attributes improves the understandability of the considered model and allows one to discover the relationship between attributes and the class variable. Secondly, it helps to devise approaches with better generalization and larger predictive power [20]. In the case of some classification methods, feature selection is an integral element of learning the model; for example, in tree-based methods, relevant attributes are chosen during the building of the tree. Another solution is using regularization techniques [18], such as least absolute shrinkage and selection operator (LASSO) regularization, which ensure sparsity in the parameter vector and allow one to find attributes influencing the class variable.

In this study, we explored the possibility of increasing the accuracy in predicting eye colour. To this end, we adopted the following strategies: (1) quantitative characterization of samples using high-quality images of the iris analysed with Digital Iris Analysis Tool (DIAT) software; (2) whole-exome sequencing (WES)-based identification of new potential predictors in a group of 150 phenotypically diverse Polish samples using the HyperLasso method and regression-based single-SNP association testing; (3) predictive modelling conducted based on the literature and WES-identified markers, using various machine learning algorithms and independent sets of samples in order to find the most accurate method for eye colour in a moderate dimensional dataset.

## Materials and methods

### Sample collection and DNA extraction

The study cohort consisted of 999 unrelated individuals (673 males and 326 females), collected together within the NEXT project, funded by the National Centre for Research and Development, grant number DOB-BIO7/17/01/2015. The study was approved by the Ethics Committee of the Jagiellonian University in Kraków (decision no. KBET/122/6120/11/2016), and all volunteers gave written informed consent prior to their inclusion in the study. Recruitment of the participants was carried out in the Police Academy in Szczytno.

Whole blood was collected from the volunteers and subjected to DNA extraction using the PrepFiler Express™ Forensic DNA Extraction Kit (Thermo Fisher Scientific) according to the manufacturer’s protocol. Quantification of the extracted samples was performed using the Quantifiler™ Human DNA Quantification Kit or the Plexor® HY System.

### Phenotype assessment

Phenotyping of the investigated samples for eye colour was conducted in two independent ways: quantitative measurements, used for identification of new SNP candidates from WES analysis, and categorization, used at the predictive modelling stage. The evaluation was performed based on collected high-resolution photographic documentation. Photos of both eyes were taken in identical conditions for all volunteers using a Nikon D5300 camera with an R1C1 Wireless Close-up Speedlight System (Nikon, Tokyo, Japan). Images of the iris were taken from a distance of about 20–30 cm, with the following settings: shutter speed 1/125, aperture *f*/22, ISO 200, flash A = 1/8, B = 1/4. Eye colour was classified into 3 categories: blue, intermediate (green, green-hazel) and brown. Classification was performed based on photographic documentation of both irises, by one assessor. The assignment to a specific category was carried out in two independent rounds of classification, or three, when there was an inconsistency between the first and the second round of assessment. The second approach consisted in an objective, quantitative characterization of eye colouration. Eye colour quantitative evaluation was conducted based on high-quality images and with DIAT software [21]. Blue and brown pixels in the area of the iris are counted and the Pixel Index of Eye (PIE score) is calculated as a measure of eye pigmentation. The PIE score ranges between − 1 (which corresponds to perfectly brown eye colour) and 1 (which corresponds to perfectly blue eyes). Additional information that was used in statistical analyses included age and sex. The studied group was divided into two sets: the discovery cohort consisted of 150 phenotypically diverse samples, used for candidate markers selection based on WES analysis, and the predictive modelling cohort consisted of the remaining 849 samples, used to develop and evaluate predictive models.

### Whole-exome sequencing of the discovery cohort

Exonic sequences (66 Mbp) enriched in regulatory regions of > 160 loci with a known association with human appearance traits (1.5 Mbp) extracted from Nencki Genomics Database and FANTOM [22, 23] were sequenced and bioinformatically analysed in the group of 150 carefully selected, phenotypically diverse individuals, as described in detail in [14]. As a result, genetic data for 77,485 SNPs with less than 20% of missing data and global minor allele frequency ≥ 5% were extracted for further statistical analyses.

### Selection of potential DNA predictors

Taking into consideration the high importance of precise phenotype characterization and the fact that the studied trait exhibits continuous distribution, WES-based marker selection for eye colour was performed based on quantitative measurements, which provide an objective and accurate trait description. Two different statistical approaches were applied for candidate marker selection. As it is still the most common concept, especially when handling large numbers of tested variables, single marker testing was applied. In order to increase the chance of identification of powerful predictors, we decided to set the suggestive threshold of *P*-value < 1 × 10^−4^ for candidate SNP selection. Because of the character of the data, linear regression for quantitatively described eye colour was used. Results were adjusted for age and sex. In addition, the HyperLasso method (https://www.ebi.ac.uk/projects/BARGEN) was applied as an alternative approach for feature selection. It is a highly attractive method that addresses the computational challenge of simultaneous SNP analysis from large-scale experiments. It is a model selection method that utilizes a Bayesian-based penalized maximum likelihood approach, which can handle high-dimensional inputs [24]. Various penalty and shape parameters were tested. The best ones were selected empirically, based on the assumption that the model should consist of a reasonable number of predictors (*p*), i.e. 0 < *p* < 100. All newly selected candidate SNP markers were subjected to linkage disequilibrium (LD) pruning and one SNP from each LD block (r^2^ > 0.7) was kept for further analyses. LD analysis was conducted using PLINK 1.9, while remaining analyses were conducted with R v3.5.2 using ‘ordinal’ package and ‘HyperLasso’ code. Since many variants associated with human pigmentation traits have already been identified, an intensive literature review was conducted and 114 SNP markers, previously correlated with pigmentation in general, were selected for further statistical analyses (Supplementary Information; Table S4).

### Targeted sequencing of DNA candidates

Genetic data for 141 DNA variants in a population of 849 individuals were collected using targeted high-throughput DNA sequencing with Ion AmpliSeq™ technology and an Ion S5™ or Ion Proton™ platform. Two independent Ion AmpliSeq™ custom panels were designed using Ion AmpliSeq™ Designer tool (https://www.ampliseq.com/https://www.ampliseq.com) with Thermo Fisher Scientific support, and covered DNA markers for various human appearance traits, including pigmentation, hair morphology, hair greying, earlobe, monobrow and other traits investigated within the NEXT project. Because of technical problems, four SNPs were replaced by SNPs in LD (rs2004775—> rs60247077 in *RBFOX1*, rs7762830—> rs743589 in *MYB*, rs224223—> rs224219 in *MEFV* and rs12052928—> rs9636495 in *ANKRD36*). DNA libraries were prepared manually and sequenced as described previously [11]. Missing SNP data were at the level of 0.2% and were imputed using the ‘missForest’ method in R v3.5.2 (with a total number of trees equal to 500).

### Predictive modelling

All candidate SNP variants selected from the literature and WES analysis were used in prediction modelling except four variants (rs1800414 in *OCA2*, and rs3212355, rs312262906 and rs201326893 in *MC1R*), which were monomorphic in our dataset and were excluded from statistical analyses. The final list of variables also included age and sex. Various machine learning algorithms were evaluated for the most accurate model development. Models were developed to predict eye colour categorized in three classes.

#### Regression models

Marker selection for regression models was performed by the forward selection method with two classical statistical approaches, the Akaike Information Criterion (AIC) [25] and the Bayesian Information Criterion (BIC) [26]. They are used to find a trade-off between the goodness of fit of a model and its complexity and are suitable in the case where *p* < *n* (*p*-total number of variables, *n*-total number of cases). In order to determine their robustness, two additional regression models, i.e. (1) developed using only one marker—the most important one—chosen in the first round of SNP selection (LOG 1-STEP), and (2) using all the analysed in this study SNPs (LOG FULL), were added for comparison purposes. In addition, we used LASSO, in which the penalized log-likelihood function is considered [27]. This regularized regression method is particularly popular in high-dimensional statistical data analysis. LASSO shrinks some coefficients of the model to zero, and therefore, it can be regarded as a feature selection method. Analyses were carried out using RStudio (v 1.1.456) and the *glmnet* package.

#### Other machine learning algorithms

The performance of seven additional machine learning algorithms was also tested. They included Classification and Regression Trees (TREE), Random Forests (RF), Extreme Gradient Boosting (XGB), Multivariate Adaptive Regression Splines (MARS), Neural Network (NN), Support Vector Machine (SVM) and Naïve Bayes (NB). A random naive classifier, which assigned observations randomly to classes according to apriori probabilities (Naive), was used as a benchmark. Default settings were applied to tested algorithms. Feature selection for TREE, RF, XGB and MARS algorithms is embedded in the learning algorithm, i.e. relevant features were selected during fitting the models. Analyses were carried out using R (v 4.0.3) and the following packages: *infotheo*, *missForest*, *cvTools*, *rpart*, *randomForest*, *xgboost*, *glmnet*, *class*, *ROCR*, *earth*, *nnet* and *caret*. All tested algorithms with their abbreviations used in the text are listed in Table 1.

**Table 1:** List of the machine learning approaches evaluated for eye colour prediction

Algorithm/model	Abbreviation
Random classifier	Naive
Logistic/multinomial regression with LASSO regularization	LOG REG
Logistic/multinomial regression with AIC-based model selection	LOG AIC
Logistic/multinomial regression with BIC-based model selection	LOG BIC
Logistic/multinomial regression with 1 step (1 SNP)	LOG 1-STEP
Logistic/multinomial regression model with all SNPs	LOG FULL
Classification and Regression Tree	TREE
Random forest	RF
Extreme gradient boosting	XGB
Multivariate and adaptive regression splines	MARS
Neural networks	NN
Support vector machine	SVM
Naïve Bayes	NB

#### Evaluation of the models’ performance

In order to assess the performance of the fitted models, the predictive modelling cohort was divided into training (70%) and testing (30%) datasets, which is a commonly used strategy, close to optimal for reasonable sized datasets (n ≥ 100) with strong signals (≥ 85% accuracy) [28]. Additionally, to reduce randomness of data splitting, the process was repeated 100 times, with seed numbers 1–100, using the function set.seed in R. The final performance of the models was determined based on the results collected from 100 testing sets, by calculation of the mean values of the following measures:

- AUC — area under the ROC curve — expressing the overall prediction accuracy and ranges between 0.5, which corresponds to random classification, and 1.0, which corresponds to perfect classification
- Accuracy (acc.) — the percentage of individuals correctly classified into a specific category over the total number of individuals in the analysis, expressed by the equation:
  $$Accuracy=\frac{True\;Positives+True\;Negatives}{True\;Positives+True\;Negatives+False\;Positives+False\;Negatives}$$
- Sensitivity (sens.) — true positive rate, expressed by the equation:
  $$Sensitivity=\frac{True\;Positives}{True\;Positives+False\;Negatives}$$
- Specificity (spec.) — true negative rate, expressed by the equation:
  $$Specificity=\frac{True\;Negatives}{True\;Negatives+False\;Positives}$$

We calculated the above measures for each class (eye colour), i.e. the observations corresponding to the considered class are treated as positive examples, whereas observations corresponding to the two remaining classes are treated as negative examples.

## Results

### Characteristics of the study group

The study cohort consisted of 673 (67.4%) males and 326 (32.6%) females in the age range 19–77 years (mean = 30.6; SD = 9.0). Considering the categorized pigmentation phenotype, most individuals had blue eyes (62.8%) followed by brown (18.1%) and intermediate (15.0%). Due to difficulties in unambiguous eye colour categorization, forty-one individuals’ eye colour was not determined (4.1%) mainly due to varying degrees of heterochromia. There was no statistically significant correlation between categorized eye colour and age or sex (*P*-value = 0.349 and *P*-value = 0.582, respectively). Quantitative measurements of eye colour revealed a whole range of possible phenotypes, from PIE score =  − 1 to 1. Mean PIE score was 0.2 (SD = 0.8). The borderline statistical significance was noted for correlation between quantitatively described eye colour and age (*P*-value = 0.041) but not sex (*P*-value = 0.321). Nevertheless, although statistically significant, correlation between PIE score and age was negligible in the studied sample set (r = 0.065). Information about eye colour of the whole studied cohort, and divided into discovery and predictive modelling cohorts, is provided in Table 2.

**Table 2:** Characteristics of the study group

	Discovery cohort [N = 150]		Predictive modelling cohort [N = 849]		Total [N = 999]	
Sex						
Females	67	44.7%	259	30.5%	327	32.7%
Males	83	55.3%	590	69.5%	673	67.4%
Age						
min	19		19		19	
max	77		62		77	
mean value	31.5		30.4		30.6	
SD	10.3		8.7		9.0	
Eye colour						
Blue	76	50.7%	551	64.9%	627	62.8%
Intermediate	28	18.7%	122	14.4%	150	15.0%
Brown	42	28.0%	139	16.4%	181	18.1%
NA	4	2.7%	37	4.4%	41	4.1%
PIE score, min	− 1.0		− 1.0		− 1.0	
PIE score, max	1.0		1.0		1.0	
PIE score, mean value	0.0		0.2		0.2	
PIE score, SD	0.9		0.8		0.8	

### Selection of DNA markers

**Fig. 1:**
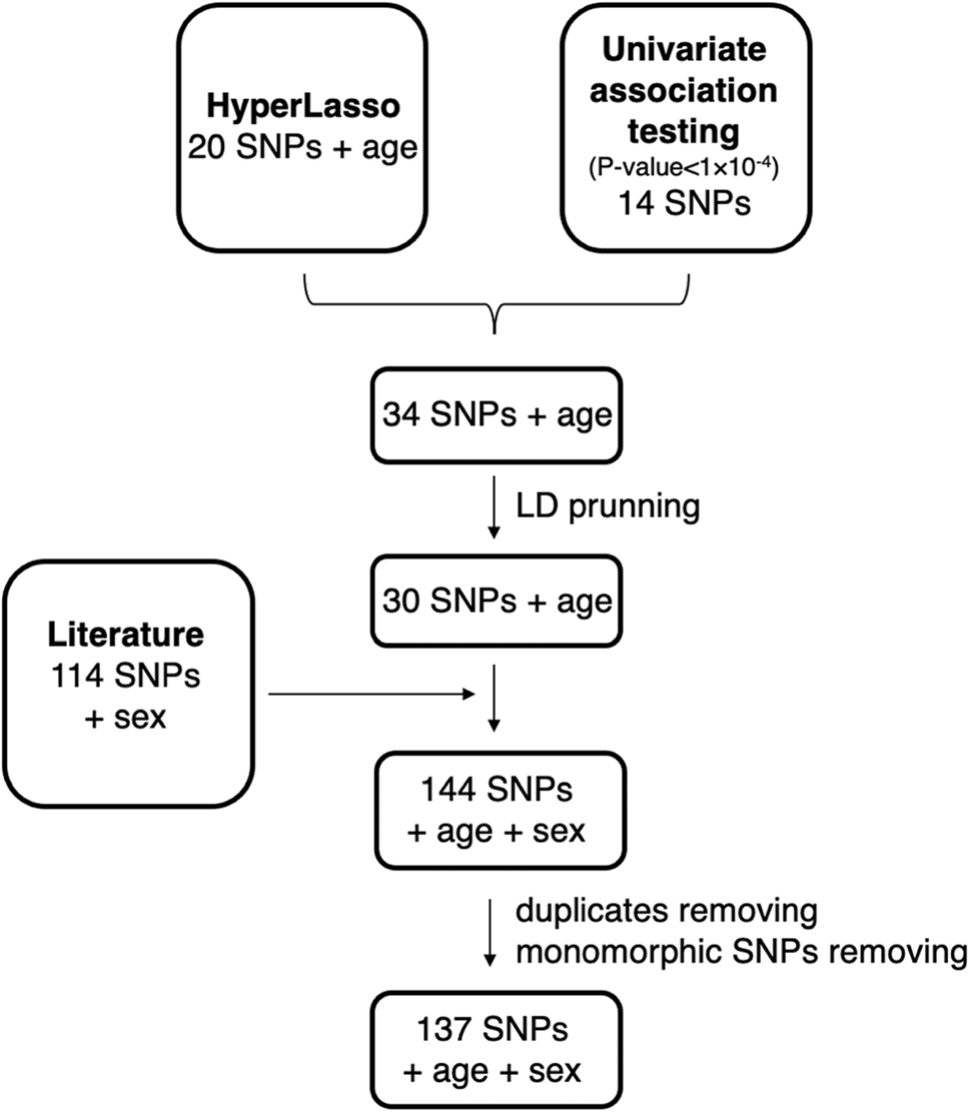
Selection of markers subjected to the predictive modelling

Univariate association testing conducted on WES data generated for 150 samples included in the discovery cohort allowed the selection of 14 candidates for eye colour (*P*-value < 1 × 10^−4^) (Supplementary Information; Table S1). The HyperLasso method identified an additional 20 candidate SNPs and age as important variables for eye colour (Supplementary Information; Table S2). Subsequent analysis of the linkage disequilibrium (Supplementary Information; Table S3) led to a set of 30 independent (r^2^ < 0.7) candidate DNA markers for eye colour. A literature search revealed a further 114 DNA markers previously associated with pigmentation (all listed in Supplementary Information; Table S4) and three SNPs (rs12896399 in *SLC24A4*, rs7495174 in *OCA2* and rs11636232 in *HERC2*) overlapped with WES-based selected variants. Overall, WES analysis discovered twenty-seven novel candidates for eye colour. As the four SNPs were monomorphic in the studied dataset, prediction modelling finally involved analysis of 137 SNPs, age and sex (Fig. 1).

### Prediction modelling using regression methods

#### Development of the models

**Fig. 2:**
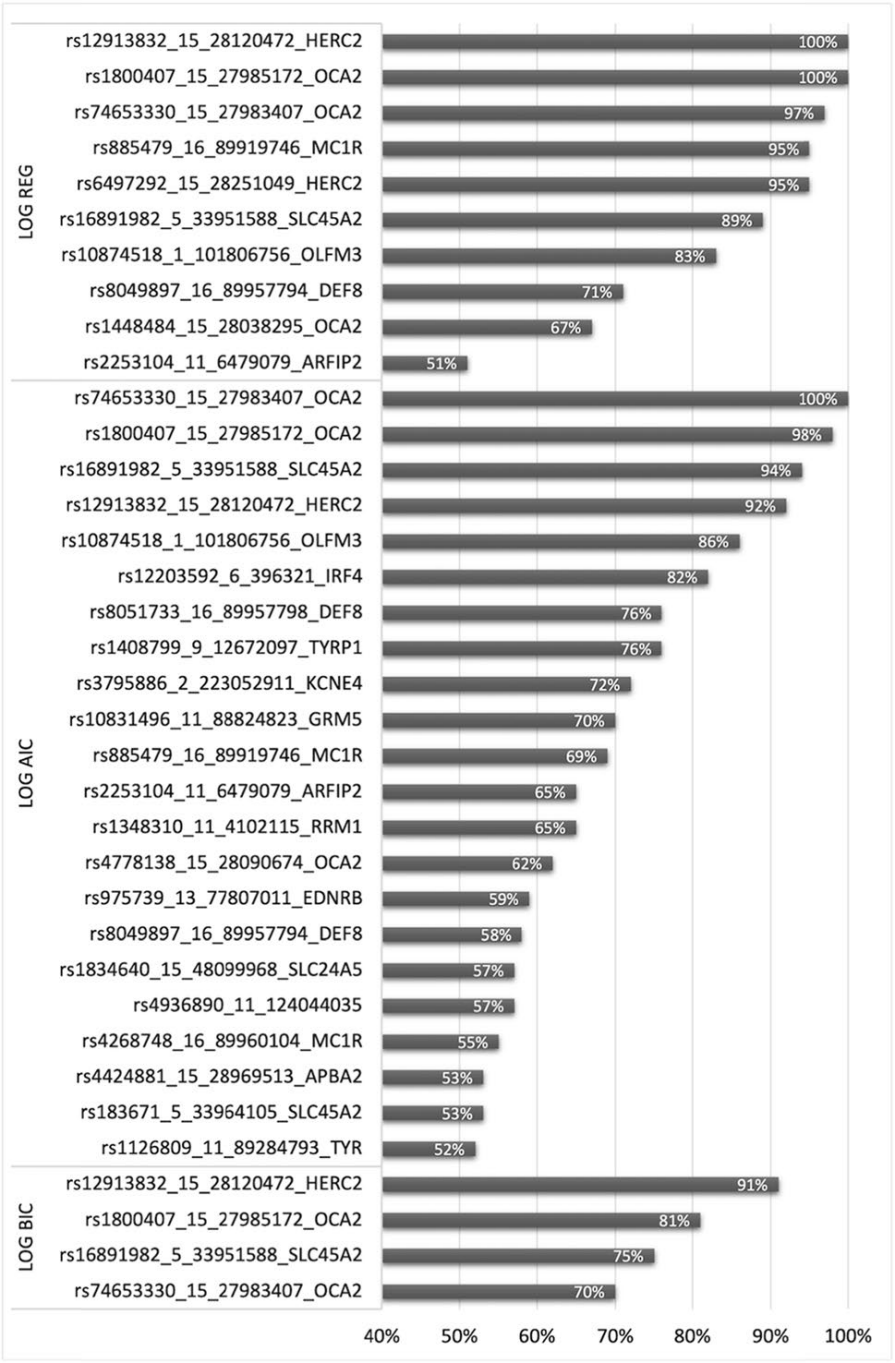
Predictive marker selected by the LASSO (LOG REG), AIC (LOG AIC) and BIC (LOG BIC) approaches for eye colour prediction. Only stable markers (selected to at least 50, i.e. 50% of models) are presented in the chart

AIC, BIC and LASSO were used for marker selection, resulting in unique sets of variables selected in each of 100 data splits. Analysis revealed that the BIC method produced the most parsimonious models while the most extensive models were developed using AIC. Further analysis showed that these extensive models contained variables that were selected for parsimonious models, which means that in many cases, markers selected by BIC were a subset of AIC- and LASSO-based developed models. The most important predictors were chosen on the basis of two criteria: (1) selected in > 50 out of 100 data splits and (2) selected by at least two selection methods. Among them, besides well-known literature predictors, was the novel marker rs2253104 in *ARFIP2*. The most important variants fulfilling both conditions are listed in Table 3. Figure 2 shows all ‘stable’ variants, i.e. those that were selected with each selection method in > 50 out of 100 data splits.

**Table 3:** The most important SNP variants selected in > 50 out of 100 data splits, by at least two of variables selection methods for regression models

SNP_ID	Chromosome position (GRCh38)	Gene	Selection method
rs10874518	1:101,806,756	*OLFM3*	LASSO, AIC
rs16891982	5:33,951,588	*SLC45A2*	LASSO, AIC, BIC
rs2253104	11:6,479,079	*ARFIP2*	LASSO, AIC
rs12913832	15:28,120,472	*HERC2*	LASSO, AIC, BIC
rs1800407	15:27,985,172	*OCA2*	LASSO, AIC, BIC
rs74653330	15:27,983,407	*OCA2*	LASSO, AIC, BIC
rs885479	16:89,919,746	*MC1R*	LASSO, AIC
rs8049897	16:89,957,794	*DEF8*	LASSO, AIC

#### Testing of models’ performance

Five different approaches were analysed in order to find the most accurate methods for regression model development. The highest prediction accuracies were achieved using the BIC and LASSO regularization methods. These values reached acc. = 0.84 and 0.85, respectively, which means that 84–85% of individuals were classified into the correct eye colour category. Slightly lower accuracies were achieved with the AIC method and for the 1-STEP model (for both acc. = 0.79), and the lowest for the LOG FULL model (acc. = 0.74). Moreover, high AUC values were noted for all categories, including intermediate eye colour (AUC = 0.85), equal for models developed with the help of the BIC and LASSO approaches. Using these methods, blue and brown eye colours were predicted with an AUC of 0.96 and 0.93–0.94, respectively. Interestingly, a 1-STEP model (which was always based on one of the key variants in the *HERC2* gene, selected as the best predictor) achieved slightly better AUC values than both AIC-based and FULL models and only marginally lower compared to BIC- and LASSO-based developed models for all eye colour categories. The differences were mostly observed for the intermediate category (AUC = 0.83 for the 1-STEP model, 0.75 for LOG AIC, 0.67 for LOG FULL and 0.85 for both LOG REG and LOG BIC), while for blue and brown eye colours, the differences were less pronounced. Most importantly, the sensitivity of intermediate eye colour prediction reached high values, especially in the case of LOG AIC: sens. = 0.40 and LOG FULL: sens. = 0.41. LOG BIC and LOG REG also showed relatively good values of 0.29 and 0.17, respectively, but not LOG 1-STEP: 0.00. At the same time, the specificity of the LOG BIC and LOG REG models was very high, reaching values of 0.96 and 0.97, respectively, while LOG FULL and LOG AIC remained reasonably high: 0.85–0.88. Detailed results of prediction performance analysis are shown in Table 4.

**Table 4:** Detailed results of predictive analysis of eye colour with various machine learning approaches

Accuracy		Naive	LOG REG	LOG AIC	LOG BIC	LOG 1-STEP	LOG FULL	TREE	RF	XGB	MARS	NN	SVM	NB
Mean		0.33	0.85	0.79	0.84	0.79	0.74	0.82	0.83	0.81	0.82	0.77	0.79	0.77
SD		0.03	0.02	0.03	0.03	0.02	0.03	0.03	0.02	0.02	0.03	0.03	0.03	0.02
AUC														
Mean	Blue	0.50	0.96	0.91	0.96	0.95	0.92	0.95	0.96	0.96	0.95	0.93	0.95	0.94
SD		0.04	0.01	0.03	0.01	0.01	0.02	0.01	0.01	0.01	0.03	0.05	0.01	0.02
Mean	Intermediate	0.51	0.85	0.75	0.85	0.83	0.67	0.82	0.84	0.82	0.80	0.77	0.82	0.80
SD		0.05	0.03	0.07	0.03	0.03	0.04	0.04	0.03	0.03	0.06	0.06	0.03	0.03
Mean	Brown	0.49	0.94	0.88	0.93	0.91	0.82	0.91	0.92	0.91	0.91	0.87	0.91	0.89
SD		0.05	0.02	0.07	0.02	0.01	0.03	0.03	0.01	0.02	0.03	0.08	0.02	0.02
Sensitivity														
Mean	Blue	0.17	0.96	0.93	0.97	0.96	0.86	0.96	0.96	0.97	0.96	0.95	0.96	0.94
SD		0.03	0.01	0.03	0.01	0.01	0.04	0.01	0.01	0.01	0.02	0.02	0.01	0.02
Mean	Intermediate	0.17	0.17	0.40	0.29	0.00	0.41	0.35	0.11	0.34	0.39	0.18	0.10	0.16
SD		0.06	0.09	0.09	0.13	0.00	0.08	0.11	0.05	0.09	0.09	0.24	0.09	0.16
Mean	Brown	0.16	0.62	0.59	0.76	0.37	0.56	0.62	0.42	0.61	0.63	0.55	0.46	0.67
SD		0.06	0.17	0.09	0.19	0.42	0.09	0.11	0.09	0.08	0.10	0.37	0.16	0.18
Specificity														
Mean	Blue	0.84	0.94	0.85	0.93	0.94	0.88	0.93	0.94	0.93	0.92	0.82	0.88	0.79
SD		0.04	0.02	0.05	0.03	0.02	0.04	0.03	0.03	0.03	0.04	0.14	0.04	0.06
Mean	Intermediate	0.83	0.97	0.88	0.96	1.00	0.85	0.91	0.98	0.92	0.91	0.94	0.97	0.95
SD		0.03	0.02	0.03	0.03	0.00	0.03	0.02	0.01	0.02	0.03	0.08	0.03	0.04
Mean	Brown	0.83	0.93	0.93	0.90	0.94	0.88	0.90	0.94	0.90	0.92	0.89	0.92	0.86
SD		0.02	0.03	0.02	0.03	0.09	0.03	0.03	0.02	0.02	0.02	0.07	0.03	0.05

### Prediction modelling using other machine learning methods

In search of improvements in predicting eye colour, especially the intermediate category, several more advanced machine learning approaches were evaluated. Results showed that all tested methods revealed quite similar performances as measured by accuracy and AUC parameters. Among them, the highest accuracies were found for RF and MARS (acc. = 0.83 and 0.82, respectively). The poorest accuracies were achieved by NB and NN (acc. = 0.77). When it comes to AUC, the highest values were estimated for RF for all eye colour categories. Nevertheless, all these methods (RF, XGB, MARS and SVM) gave similar AUC and accuracy outcomes.

However, sensitivity and specificity values varied substantially among the tested methods. The lowest sensitivity values were estimated for RF (although the overall prediction accuracy estimated for this method was relatively high) and SVM, followed by NB and NN. The greatest differences were found for intermediate eye colour, which was particularly interesting. The highest sensitivity values were estimated for MARS (sens. = 0.39), TREE (sens. = 0.35) and XGB (sens. = 0.34), while they were significantly lower for NN (sens. = 0.18), NB (sens. = 0.16), RF (sens. = 0.11) and SVM (sens. = 0.10). On the other hand, specificity values were the highest for RF and SVM (spec. = 0.98 and 0.97, respectively), followed by NB and NN (spec. = 0.95 and 0.94) then TREE, XGB and MARS (spec. = 0.91–0.92). Details of prediction modelling analysis conducted using advanced machine learning algorithms are shown in Table 4.

## Discussion

Accuracy of phenotype prediction from genetic data is essential for the successful application of predictive methods in biomedical studies including anthropology, paleogenetics and forensics [29]. Several factors determine good accuracy of DNA-based predictive methods, including high heritability of a trait, identification of appropriate predictors and selection of the best mathematical approach to model development. Even highly heritable traits are often difficult to predict, due to polygenicity, epistasis, and allelic and locus heterogeneity. In this study, we used quantitative assessment of eye colour phenotypes and whole exome/regulome sequencing to identify additional predictors, and additionally, we verified multiple machine learning methods to assess their impact on prediction accuracy, focusing especially on more complex intermediate phenotypes. The studied cohort of Polish individuals shows a relatively large diversity of pigmentation phenotype compared to some other European populations, which makes it useful for studying the genetics of pigmentation traits. Objective phenotyping of eye colour for finding new loci provided quantitative measurements. The analysis using DIAT software [21] confirmed that the calculated PIE score reflecting the ratio of blue to brown pixels highly correlates with human evaluation of eye colour (Spearman correlation =  − 0.82, *P*-value = 5.46 × 10^−233^). Using the single-SNP association testing of the WES/regulome data under *P*-value < 1 × 10^−4^, and the HyperLasso algorithm, which aimed to select the subset of SNPs that best predicted the trait under study simultaneously controlling the type I error of the selected variants [24], we identified 34 SNPs and age as important factors for eye colour prediction. In the next step, we moved directly to the extensive predictive modelling.

A large number of algorithms have been developed to deal with a variety of increasingly demanding and computationally challenging data analyses. Analysis of AIC, BIC and LASSO methods of marker selection conducted in this study revealed that all of them are robust, since they produced models with better performance compared to models without any selection method applied (i.e. LOG FULL). We confirmed that BIC, which more heavily penalizes the introduction of additional variables, produced the most parsimonious models. Together with BIC, LASSO yielded models with the best predictive performance. Interestingly, focusing on SNPs selected by at least two out of three feature selection methods and in at least 50% of data splits, we found the well-known pigmentation markers and the intronic variant rs2253104 in *ARFIP2*, newly identified by HyperLasso (Table 3). *ARFIP2* is located on 11p15.4 and encodes for ADP-ribosylation factor-interacting protein 2 (ARFIP2), which is highly expressed in various tissues. This protein has been shown to be involved in several cellular processes and signalling pathways. They include Rac1-mediated signalling, triggering actin polymerization [30], which in melanocytes is involved in dendrites formation and therefore the transport of melanosomes to keratinocytes [31]. Also, ARFIP2 has been shown to negatively regulate NF-κB signalling [32], inducing MITF expression, one of the most important melanogenesis regulators [33]. Interestingly, *ARFIP2* was among the downregulated genes in human melanoma cells treated with arbutin [34], which is a known inhibitor of melanin biosynthesis used in cosmetology for skin whitening [35]. Therefore, although there is no evidence that ARFIP2 is directly involved in melanogenesis, it is possible that it may be engaged in indirect regulation of pigmentation-related genes. It has been speculated that the missing heritability of many complex traits can be explained by gene action outside the core pathways [36]. So far, rs2253104 in *ARFIP2* has been associated with lung cancer [37]. Rs2253104 in *ARFIP2* was selected in 65% of LOG AIC models and in 51% of LOG REG models, therefore more frequently than, e.g. rs12203592 in *IRF4* or rs1408799 in *TYRP1* (LOG REG), the other well-established eye colour predictors (Fig. 1). Nevertheless, as the univariate association analysis did not reveal statistically significant association of rs2253104 with eye colour either in discovery or predictive modelling cohort, its effect appears to be very complex and the direction of the effect difficult to interpret. Therefore, further studies are needed to support our hypothesis about the potential role of this variant and better understand this effect. The nonsynonymous *OCA2* rs74653330 variant, which was very often selected by all three (AIC, BIC, REG) methods, also deserves more attention. The research by Yuasa et al. showed a north–south geographic gradient of the rarer T allele, which was interpreted as a possible case of adaptive evolution [38]. Indeed, it has been suggested that this *OCAC2* variant is responsible for reduced efficiency of melanogenesis [39] and thus lighter pigmentation, which is preferred in areas with lower ultraviolet radiation content. Notably, the T-allele was also found to have a measurable effect on normal eye colour variation in Scandinavian samples [40, 41]. The incidence of the minor T allele in the Scandinavian population was 0.005 and this variant was not present in the Italian and Portuguese populations [40]. In our population, the derived T allele was observed 10 times in 999 individuals, in the heterozygous genotypes. Our study confirms importance of rs74653330 for eye colour prediction and further indicates that allelic heterogeneity altogether with the population-specific differences in allele frequencies may be important factors in predictive DNA analysis. Other SNPs for eye colour prediction included three out of six variants implemented in the IrisPlex model: rs12913832 (*HERC2*), rs1800407 (*OCA2*) and rs1689182 (*SLC45A2*) [16, 42] as well as others, previously associated with eye patterning (rs10874518, *OLFM3*; [43]), or other pigmentation traits (rs885479; *MC1R*, rs8049897, *DEF8*; [44]) (Table 3).

Importantly, the accuracy of predicting intermediate eye colour achieved a high level (e.g. regression model developed with BIC approach or with LASSO regularization: AUC = 0.85), higher than reported for IrisPlex [16] and Snipper [45], the two most widely used eye colour predictive tools. In data analysed here, the sensitivity of intermediate eye colour prediction was also better (LOG BIC sens. = 0.29) compared to the results obtained with the original IrisPlex model (sens. = 0.00). In previous research, a significant increase in the sensitivity of intermediate eye colour prediction was achieved due to additional variation in the *HERC2* gene included in the predictive model. The positive effect was, however, reversible, since the addition of other *HERC2* variants decreased the ability of the model to predict intermediate eye colours [45]. A small increase in accuracy of intermediate eye colour was also reported in a study that involved genetic interactions [46].

Besides classical regression, several more advanced machine learning algorithms were evaluated. The study demonstrated that advanced machine learning methods showed even higher sensitivity values of intermediate eye colour prediction (i.e. TREE, XGB and MARS with sens._interm._ = 0.34–0.39); however, a slightly reduced sensitivity of brown eye colour prediction was observed for these models when compared to the regression model. It is well known that more advanced machine learning methods may better cope with recognition of complex phenotypes, including intermediate eye colour, due to the ability to identify possible nonlinear dependencies between variables, such as interactions. Nevertheless, while some advanced methods were found to demonstrate increased sensitivity or specificity in predicting certain categories, none of these approaches outperformed the regression method developed following prior features selection using BIC or LASSO, when AUC or accuracy metrics were compared. Moreover, differences between the tested methods were modest. These results suggest that more sophisticated learning algorithms may need larger datasets to demonstrate their superiority and do not reveal their potential in low- and medium-dimensional data. Also, a systematic review [47] of logistic regression and other machine learning methods (among which the most common were classification trees, random forests, artificial neural networks and support vector machines) showed that in the group of low risk of biased study, no performance benefit of machine learning over logistic regression methods was reported for clinical prediction models. Further, evaluation of deep learning methods (multilayer perceptron and convolutional neural networks) conducted on high-dimensional data (~ 100 k individuals and ~ 500 k SNPs) did not provide any proof that these methods outperform simple linear methods and improve complex human trait prediction by a sizeable margin [48]. Although our analysis did not involve advanced machine learning hyperparameters tuning aimed at improving the obtained prediction accuracies, there is evidence in the literature that such tuning may still not be helpful for significantly improving accuracy [49]. Nevertheless, it was found that the superiority of the advanced ML approaches (random forests) depends on the dataset and tends to be more pronounced for an increasing number of analysed features or an increase in the ratio of the number of features to the number of cases [50]. Indeed, it has been shown that some of the advanced algorithms can be very successful in predicting complex traits if applied to very high-dimensional data [51]. It is also worth noting that advanced machine learning methods outperform basic linear regression in age prediction using DNA methylation data. In the evaluation of 17 different machine learning approaches performed by Aliferi et al., the support vector machine with the polynomial function method was chosen as highly robust, generalizable and the best-performing modelling approach [52], as it was in another previous study [53]. Also, neural networks (e.g. [54]) and random forest regression [55] were successfully applied to accurate human age prediction. This demonstrates the superiority of some ML approaches over classical regression methods in data with observed nonlinear correlation effects and also suggests a possible dependence of ML methods’ efficiency on the data type: discrete for SNP vs. quantitative for DNA methylation.

In summary, whole-exome sequencing of 150 individuals has allowed identification of 27 DNA variants that are relevant for eye colour prediction which have not been reported before in pigmentation predictive studies. Besides well-known pigmentation-associated variants, rs2253104 in *ARFIP2* was selected by at least two different feature selection methods for regression predictive models, which turned out to be the most accurate. None of the sophisticated machine learning algorithms outperformed the overall prediction accuracy of regression models developed following prior features selection using BIC or LASSO regularization, indicating that medium-dimensional data does not use the whole potential of these more advanced algorithms.

## Supplementary information

Below is the link to the electronic supplementary material.

Supplementary file1 (DOCX 54.2 KB)

## Data Availability

Raw and additional data are available upon request.
