# Supplementary material for: Searching for improvements in predicting human eye colour from DNA
Source: Int J Legal Med. 2021 Jul 14;135(6):2175–87. doi: 10.1007/s00414-021-02645-5 (PMC8523394; doi:10.1007/s00414-021-02645-5)
Supplement: Supplementary file 1 — Supplementary file1 (DOCX 54.2 KB) [file 414_2021_2645_MOESM1_ESM.docx]

**Supplementary Information**

**Searching for improvements in predicting human eye colour from DNA**

*International Journal of Legal Medicine*

Magdalena Kukla-Bartoszek*, Paweł Teisseyre, Ewelina Pośpiech, Joanna Karłowska-Pik, Piotr Zieliński, Anna Woźniak, Michał Boroń, Michał Dąbrowski, Magdalena Zubańska, Agata Jarosz, Rafał Płoski, Tomasz Grzybowski, Magdalena Spólnicka, Jan Mielniczuk, Wojciech Branicki^*^

*Corresponding authors:

Wojciech Branicki

Malopolska Centre of Biotechnology of the Jagiellonian University, Kraków, Poland

Central Forensic Laboratory of the Police, Warsaw, Poland

e-mail: wojciech.branicki@uj.edu.pl

Magdalena Kukla-Bartoszek

Faculty of Biochemistry, Biophysics and Biotechnology, Jagiellonian University, Kraków, Poland

Malopolska Centre of Biotechnology of the Jagiellonian University, Kraków, Poland

e-mail: magda.kukla@uj.edu.pl

**Supplementary Table S1** Candidate markers identified with single SNP-testing in whole-exome data.

| **No.** | **Variable ID** | **Chromosome position GRCh38** | **Gene** | **Discovery [N=150]** | | | |
| --- | --- | --- | --- | --- | --- | --- | --- |
|  |  |  |  | **MA** | **fMA** | **Beta** | ***P*-value** |
| 1 | rs2288656 | 2:127637301 | *MYO7B* | A | 0.245 | -0.43 | 9.36×10^-5^ |
| 2 | rs3795886 | 2:223052911 | *KCNE4* | C | 0.297 | -0.43 | 5.46×10^-5^ |
| 3 | rs3734421 | 6:168608265 | *SMOC2* | T | 0.163 | 0.59 | 3.17×10^-5^ |
| 4 | rs41300568 | 10:127052853 | *DOCK1* | T | 0.067 | 0.77 | 1.83×10^-5^ |
| 5 | rs1348310 | 11:4102115 | *RRM1* | C | 0.080 | 0.77 | 9.07×10^-5^ |
| 6 | rs2234593 | 11:32392787 | *WT1* | T | 0.058 | -0.91 | 7.98×10^-5^ |
| 7 | rs7164220 | 15:28096463 | *OCA2* | C | 0.147 | -0.70 | 1.51×10^-6^ |
| 8 | rs7495174 | 15:28099092 | *OCA2* | G | 0.060 | -1.07 | 3.04×10^-7^ |
| 9 | rs11636232 | 15:28141480 | *HERC2* | T | 0.380 | 0.49 | 7.73×10^-7^ |
| 10 | rs61756152 | 15:28167726 | *HERC2* | A | 0.070 | -0.97 | 6.77×10^-7^ |
| 11 | rs2240202 | 15:28265749 | *HERC2* | A | 0.050 | -1.00 | 1.16×10^-5^ |
| 12 | rs2004775 | 16:5239464 | *RBFOX1* | A | 0.347 | 0.50 | 6.16×10^-6^ |
| 13 | rs2836016 | 21:37840718 | *KCNJ6* | G | 0.477 | -0.42 | 3.03×10^-5^ |
| 14 | rs60600671 | 22:38109259 | *BAIAP2L2* | G | 0.103 | 0.65 | 6.21×10^-5^ |

**Supplementary Table S2** Candidate markers identified with the HyperLasso algorithm in whole-exome data.

| **No.** | **Variable ID** | **Chromosome position GRCh38** | **Gene** | **Discovery [N=150]** | | |
| --- | --- | --- | --- | --- | --- | --- |
|  |  |  |  | **MA** | **fMA** | **Effect size** |
| 1 | rs7542508 | 1:236736718 | *ACTN2* | G | 0.403 | 6.53×10^-6^ |
| 2 | rs918949 | 2:217809974 | *TNS1* | C | 0.403 | 5.99×10^-9^ |
| 3 | rs2571445 | 2:217818431 | *TNS1* | A | 0.398 | 2.48×10^-8^ |
| 4 | rs1468542 | 3:45737644 | *SACM1L* | A | 0.413 | 1.63×10^-6^ |
| 5 | rs3821751 | 3:185048604 | *VPS8* | G | 0.480 | -1.05×10^-4^ |
| 6 | rs7680528 | 4:26272652 | *RBPJ* | T | 0.460 | 0.188 |
| 7 | rs3812036 | 5:177386403 | *SLC34A1* | T | 0.253 | -1.63×10^-6^ |
| 8 | rs2253104 | 11:6479079 | *ARFIP2* | A | 0.473 | 0.185 |
| 9 | rs56262049 | 13:42070569 | *DGKH* | C | 0.233 | 2.62×10^-5^ |
| 10 | rs12896399 | 14:92307319 | *LOC105370627* | T | 0.393 | 6.53×10^-6^ |
| 11 | rs35983729 | 14:92321417 | *SLC24A4* | G | 0.393 | 6.53×10^-6^ |
| 12 | rs4144266 | 14:92322861 | *SLC24A4* | G | 0.393 | 6.53×10^-6^ |
| 13 | rs33657 | 16:11875867 | *GSPT1* | C | 0.433 | 2.62×10^-5^ |
| 14 | rs7216 | 17:19675560 | *ALDH3A2* | A | 0.466 | 2.62×10^-5^ |
| 15 | rs3744198 | 17:74960029 | *HID1* | A | 0.500 | -2.62×10^-5^ |
| 16 | rs2074893 | 19:2078410 | *MOB3A* | G | 0.305 | 2.62×10^-5^ |
| 17 | rs2074894 | 19:2078489 | *MOB3A* | C | 0.293 | 2.62×10^-5^ |
| 18 | rs9682 | 22:45541269 | *FBLN1* | T | 0.432 | -0.150 |
| 19 | rs1132201 | X:45191866 | *CXorf36* | T | 0.343 | 1.05×10^-4^ |
| 20 | rs2071776 | X:47565216 | *ARAF* | T | 0.189 | -0.114 |
| 21 | Age | - | - | - | - | 5.16×10^-3^ |

**Supplementary Table S3** LD analysis of WES-based selected candidate SNPs. Only variant pairs with r^2^>0.7 are listed.

| **No.** | **SNP_A** | **Chromosome position GRCh38** | **SNP_B** | **Chromosome position GRCh38** | **r2** | **SNP included in the further analyses** |
| --- | --- | --- | --- | --- | --- | --- |
| 1 | rs918949 | 2:217809974 | rs2571445 | 2:217818431 | 1.00 | rs918949 |
| 2 | rs12896399 | 14:92307319 | rs35983729 | 14:92321417 | 1.00 | rs12896399 |
| 3 | rs12896399 | 14:92307319 | rs4144266 | 14:92322861 | 1.00 | rs12896399 |
| 4 | rs35983729 | 14:92321417 | rs4144266 | 14:92322861 | 1.00 | rs12896399 |
| 5 | rs2074893 | 19:2078410 | rs2074894 | 19:2078489 | 0.96 | rs2074893 |

**Supplementary Table S4** Candidate SNP markers selected from the literature resources.

| **No.** | **Variable ID** | **Chromosome position GRCh38** | **Gene** | **MA** | **fMA** | **References** |
| --- | --- | --- | --- | --- | --- | --- |
| 1 | rs10874518 | 1:101806756 | *OLFM3* | C | 0.353 | Larsson et al., 2011 |
| 2 | rs3002288 | 1:212953223 | *VASH2* | A | 0.454 | Zhang et al., 2013 |
| 3 | rs3768056 | 1:235744525 | *LYST* | G | 0.219 | Liu et al., 2010 |
| 4 | rs205611 | 2:74744954 | *-* | G | 0.181 | Larsson et al., 2011 |
| 5 | rs6742078 | 2:233763993 | *UGT1A* | T | 0.363 | Jacobs et al., 2013 |
| 6 | rs79693451 | 2:238387832 | *TRAF3IP1* | G | 0.069 | Larsson et al., 2011 |
| 7 | rs13140875 | 4:167936311 | *ANXA10* | C | 0.113 | Larsson et al., 2011 |
| 8 | rs12520016 | 5:6767199 | *LOC102724943* | G | 0.051 | Zhang et al., 2013 |
| 9 | rs16891982 | 5:33951588 | *SLC45A2* | C | 0.038 | Stokowski et al., 2007; Han et al., 2008; Branicki et al., 2008; Eriksson et al., 2010; Mengel-From et al., 2010 |
| 10 | rs28777 | 5:33958854 | *SLC45A2* | C | 0.034 | Han et al., 2008; Branicki et al., 2011 |
| 11 | rs26722 | 5:33963765 | *SLC45A2* | T | 0.023 | Stokowski et al., 2007, Han et al., 2008, Branicki et al., 2011 |
| 12 | rs183671 | 5:33964105 | *SLC45A2* | T | 0.035 | Stokowski et al., 2007, Liu et al., 2015 |
| 13 | rs13289 | 5:33986304 | *SLC45A2* | C | 0.426 | Graf et al., 2007; Maroñas et al., 2015 |
| 14 | rs12203592 | 6:396321 | *IRF4* | T | 0.077 | Han et al., 2008; Nan et al., 2009; Duffy et al., 2010; Eriksson et al., 2010; Liu et al., 2009; 2010; Valenzuela et al., 2010; Branicki et al., 2011; Kosiniak-Kamysz et al., 2012; Praetorius et al., 2013; Hart et al., 2013; Zhang et al, 2013; Jacobs et al, 2015; Visser et al, 2015; Kukla-Bartoszek et al., 2019 |
| 15 | rs62389424 | 6:422631 | *IRF4* | A | 0.069 | Lin et al., 2015 |
| 16 | rs4959270 | 6:457748 | *EXOC2* | A | 0.438 | Han et al., 2008; Branicki et al., 2011 |
| 17 | rs1540771 | 6:466033 | *LOC105374875* | T | 0.455 | Sulem et al., 2007; Han et al., 2008; Branicki et al., 2011 |
| 18 | rs2110015 | 7:17773115 | *SNX13* | C | 0.390 | Martin et al., 2017 |
| 19 | rs10235789 | 7:84027484 | *SEMA3A* | C | 0.479 | Larsson et al., 2011 |
| 20 | rs2373391 | 7:88819986 | *ZNF804B* | A | 0.240 | Rawofi et al., 2017 |
| 21 | rs2093835 | 9:2229335 | *LOC107987043* | G | 0.147 | Martin et al., 2017 |
| 22 | rs7866411 | 9:2475084 | *LOC101930053* | G | 0.317 | Martin et al., 2017 |
| 23 | rs1408799 | 9:12672097 | *TYRP1* | T | 0.307 | Sulem et al., 2008; Pośpiech et al., 2011; 2014 |
| 24 | rs683 | 9:12709305 | *TYRP1* | C | 0.348 | Alonso et al., 2008; Valenzuela et al., 2010; Branicki et al., 2011 |
| 25 | rs10756819 | 9:16858086 | *BNC2* | G | 0.398 | Jacobs et al, 2013; Visser et al., 2014 |
| 26 | rs2153271 | 9:16864523 | *BNC2* | C | 0.471 | Eriksson et al., 2010; Hernando et al., 2018 |
| 27 | rs12350739 | 9:16885019 | *BNC2* | G | 0.479 | Visser et al., 2014, Hysi et al., 2018 |
| 28 | rs35264875 | 11:69078931 | *TPCN2* | T | 0.236 | Sulem et al., 2008 |
| 29 | rs3829241 | 11:69087895 | *TPCN2* | A | 0.380 | Maroñas et al., 2015; Morgan et al., 2018 |
| 30 | rs72930659 | 11:69105375 | *TPCN2* | T | 0.187 | Lin et al., 2015 |
| 31 | rs10831496 | 11:88824823 | *GRM5* | G | 0.341 | Nan et al., 2009; Beleza et al., 2013 |
| 32 | rs1042602 | 11:89178528 | *TYR* | A | 0.305 | Stokowski et al., 2007; Sulem et al., 2007 |
| 33 | rs1847134 | 11:89272085 | *TYR* | C | 0.285 | Eriksson et al., 2010; |
| 34 | rs1393350 | 11:89277878 | *TYR* | A | 0.240 | Eriksson et al., 2010; Liu et al., 2009; Branicki et al., 2011 |
| 35 | rs1126809 | 11:89284793 | *TYR* | A | 0.248 | Zhang et al., 2013 |
| 36 | rs4936890 | 11:124044035 | *-* | A | 0.470 | Walsh et al., 2017 |
| 37 | rs10777129 | 12:88567936 | *KITLG* | A | 0.061 | Mengel-From et al., 2009 |
| 38 | rs12821256 | 12:88934558 | *KITLG* | C | 0.087 | Sulem et al., 2007; Zhang et al., 2013 |
| 39 | rs975739 | 13:77807011 | *EDNRB* | G | 0.395 | Zhang et al., 2013 |
| 40 | rs3782974 | 13:94440642 | *DCT* | T | 0.134 | Lao et al., 2007; Walsh et al., 2017 |
| 41 | rs4900109 | 14:92297047 | *SLC32A4* | T | 0.415 | Larsson et al., 2011 |
| 42 | rs12896399 | 14:92307319 | *SLC24A4* | T | 0.413 | Eriksson et al., 2010; Zhang et al., 2013; Liu et al., 2009; Sulem et al., 2007 |
| 43 | rs4904868 | 14:92314657 | *-* | T | 0.484 | Eriksson et al., 2010; Söchtig et al., 2015 |
| 44 | rs8014907 | 14:92333660 | *SLC24A4* | T | 0.168 | Lin et al., 2015 |
| 45 | rs2402130 | 14:92334859 | *SLC24A4* | G | 0.168 | Valenzuela et al., 2010; Branicki et al., 2011 |
| 46 | rs17128291 | 14:92416482 | *SLC24A4* | G | 0.153 | Liu et al., 2015; Walsh et al., 2017 |
| 47 | rs1545397 | 15:27942626 | *OCA2* | T | 0.081 | Hart et al., 2013; Walsh et al., 2017 |
| 48 | rs7173419 | 15:27951675 | *OCA2* | T | 0.243 | Zhang et al., 2013 |
| 49 | rs1800414 | 15:27951891 | *OCA2* | 0 | 0.000 | Edwards et al., 2010; Valenzuela et al., 2010; Walsh et al., 2017 |
| 50 | rs74653330 | 15:27983407 | *OCA2* | T | 0.005 | Andersen et al., 2016 |
| 51 | rs121918166 | 15:27985101 | *OCA2* | T | 0.001 | Andersen et al., 2016 |
| 52 | rs1800407 | 15:27985172 | *OCA2* | T | 0.057 | Liu et al., 2009; Mengel-From et al., 2010; Pośpiech et al., 2011; Ruiz et al., 2013 |
| 53 | rs1800401 | 15:28014907 | *OCA2* | A | 0.035 | Duffy et al., 2007; Andersen et al., 2016 |
| 54 | rs12441727 | 15:28026629 | *OCA2* | A | 0.121 | Liu et al., 2009 |
| 55 | rs4778232 | 15:28036619 | *OCA2* | T | 0.211 | Kayser et al., 2008; Ruiz et al., 2013 |
| 56 | rs1448485 | 15:28037595 | *OCA2* | T | 0.126 | Walsh et al., 2017 |
| 57 | rs1448484 | 15:28038295 | *OCA2* | G | 0.002 | Kayser et al., 2008; Liu et al., 2009 |
| 58 | rs16950821 | 15:28038361 | *OCA2* | A | 0.115 | Branicki et al., 2011 |
| 59 | rs8024968 | 15:28038543 | *OCA2* | T | 0.115 | Kayser et al., 2008; Ruiz et al., 2013 |
| 60 | rs1470608 | 15:28042975 | *OCA2* | T | 0.134 | Eriksson et al., 2010; |
| 61 | rs1375164 | 15:28046666 | *OCA2* | T | 0.202 | Duffy et al., 2007; Ruiz et al., 2013 |
| 62 | rs7174027 | 15:28083619 | *OCA2* | A | 0.112 | Han et al., 2008; Mengel From et al., 2009; Branicki et al., 2011; Söchtig et al., 2015 |
| 63 | rs4778138 | 15:28090674 | *OCA2* | G | 0.147 | Kayser et al., 2008; Eriksson et al., 2010; Branicki et al., 2011; Ruiz et al., 2013 |
| 64 | rs4778241 | 15:28093567 | *OCA2* | A | 0.192 | Kayser et al., 2008; Han et al., 2008; Sturm et al., 2008; Eiberg et al., 2008; Branicki et al., 2011; |
| 65 | rs7495174 | 15:28099092 | *OCA2* | G | 0.051 | Sulem et al., 2007; Sturm et al., 2008; Kayser et al., 2008; Lin et al., 2015; Söchtig et al., 2015 |
| 66 | rs1129038 | 15:28111713 | *HERC2* | C | 0.179 | Sturm et al., 2008; Eiberg et al., 2008; Ruiz et al, 2013; Morgan et al., 2018; |
| 67 | rs12913832 | 15:28120472 | *HERC2* | A | 0.179 | Han et al., 2008; Eiberg et al., 2008; Sturm et al., 2008; Liu et al., 2009; Eriksson et al., 2010; Zhang et al., 2013; Branicki et al., 2011; Walsh et al., 2017; Hysi et al., 2018; Morgan et al., 2018 |
| 68 | rs7183877 | 15:28120587 | *HERC2* | A | 0.060 | Eiberg et a, 2008; Sturm et al., 2008; Liu et al., 2010; Ruiz et z, 2013 |
| 69 | rs11636232 | 15:28141480 | *HERC2* | T | 0.418 | Han et al., 2008; Ruiz et al., 2013 |
| 70 | rs2238289 | 15:28208069 | *HERC2* | G | 0.104 | Mengel-From et al., 2009 |
| 71 | rs8182028 | 15:28222789 | *HERC2* | C | 0.042 | Liu et al., 2009; Walsh et al.; 2017 |
| 72 | rs6497292 | 15:28251049 | *HERC2* | G | 0.042 | Kayser et al., 2008 |
| 73 | rs16950941 | 15:28257598 | *HERC2* | A | 0.042 | Kayser et al., 2008 |
| 74 | rs79097182 | 15:28266851 | *HERC2* | T | 0.042 | Lin et al., 2015 |
| 75 | rs916977 | 15:28268218 | *HERC2* | T | 0.127 | Kayser et al., 2008 |
| 76 | rs1667394 | 15:28285036 | *HERC2* | C | 0.130 | Kayser et al., 2008 |
| 77 | rs12592730 | 15:28285213 | *HERC2* | A | 0.042 | Kayser et al., 2008 |
| 78 | rs4424881 | 15:28969513 | *APBA2* | T | 0.131 | Beleza et al., 2013 |
| 79 | rs2924567 | 15:47976289 | *SLC24A5* | T | 0.327 | Stokowski et al., 2007, Liu et al., 2015 |
| 80 | rs1834640 | 15:48099968 | *SLC24A5* | G | 0.009 | Stokowski et al., 2007 |
| 81 | rs1426654 | 15:48134287 | *SCL24A5* | G | 0.007 | Stokowski et al., 2007; Valenzuela et al., 2010; Maroñas et al., 2015; Walsh et al.; 2017 |
| 82 | rs11630290 | 15:63623587 | *HERC1* | T | 0.235 | Larsson et al., 2011 |
| 83 | rs2353688 | 16:86329448 | *-* | C | 0.015 | Lin et al., 2015 |
| 84 | rs3114908 | 16:89317317 | *ANKRD11* | T | 0.293 | Law et al., 2015; Walsh et al., 2017 |
| 85 | rs164741 | 16:89625890 | *DPEP1* | A | 0.249 | Han et al., 2008; Nan et al., 2009, Walsh et al., 2017 |
| 86 | rs12931267 | 16:89752324 | *FANCA* | G | 0.034 | Eriksson et al., 2010; Liu et al., 2015; Söchtig et al., 2015 |
| 87 | rs3212355 | 16:89917970 | *MC1R* | 0 | 0.000 | Valenzuela et al., 2010 |
| 88 | rs312262906 | 16:89919342 | *MC1R* | 0 | 0.000 | Branicki et al., 2011; Walsh et al., 2017 |
| 89 | rs1805005 | 16:89919436 | *MC1R* | T | 0.069 | Stokowski et al., 2007, Branicki et al., 2007; Valenzuela et al., 2010; Branicki et al., 2011; Liu et al., 2015; Morgan et al., 2018 |
| 90 | rs1805006 | 16:89919510 | *MC1R* | A | 0.002 | Stokowski et al., 2007; Branicki et al., 2007; Morgan et al., 2018 |
| 91 | rs2228479 | 16:89919532 | *MC1R* | A | 0.109 | Stokowski et al., 2007; Branicki et al., 2011; Liu et al., 2015 |
| 92 | rs11547464 | 16:89919683 | *MC1R* | A | 0.004 | Eriksson et al., 2010; Branicki et al., 2011; Morgan et al., 2018 |
| 93 | rs1805007 | 16:89919709 | *MC1R* | T | 0.033 | Stokowski et al., 2007, Sulem et al., 2007; Eriksson et al., 2010; Valenzuela et al., 2010; Branicki et al., 2011; Zhang et al., 2013; Liu et al., 2015; Hysi et al., 2018; Morgan et al., 2018 |
| 94 | rs201326893 | 16:89919714 | *MC1R* | 0 | 0.000 | Ibarrola-Villava et al., 2010; Branicki et al., 2011 |
| 95 | rs1110400 | 16:89919722 | *MC1R* | C | 0.016 | Stokowski et al., 2007, Branicki et al., 2011; Liu et al., 2015 |
| 96 | rs1805008 | 16:89919736 | *MC1R* | T | 0.079 | Stokowski et al., 2007; Valenzuela et al., 2010; Mengel-From et al., 2009; Branicki et al., 2011; Liu et al., 2015 |
| 97 | rs885479 | 16:89919746 | *MC1R* | A | 0.038 | Stokowski et al., 2007; Mengel-From et al., 2009; Branicki et al., 2011 |
| 98 | rs1805009 | 16:89920138 | *MC1R* | C | 0.001 | Stokowski et al., 2007, Eriksson et al., 2010; Branicki et al., 2011 |
| 99 | rs8049897 | 16:89957794 | *DEF8* | A | 0.121 | Nan et al., 2009; Eriksson et al., 2010; Walsh et al., 2017; Kukla-Bartoszek et al., 2019 |
| 100 | rs8051733 | 16:89957798 | *DEF8* | G | 0.289 | Law et al., 2015; Walsh et al., 2017 |
| 101 | rs4268748 | 16:89960104 | *DEF8* | C | 0.259 | Law et al., 2015; Walsh et al., 2017; Kukla-Bartoszek et al., 2019 |
| 102 | rs11076649 | 16:89992928 | *AFG3L1P* | G | 0.123 | Walsh et al., 2017 |
| 103 | rs333113 | 17:4497061 | *SPNS2* | C | 0.240 | Walsh et al., 2017 |
| 104 | rs9894429 | 17:81629785 | *NPLOC4* | T | 0.431 | Liu et al., 2010, Kukla-Bartoszek et al., 2019 |
| 105 | rs6059655 | 20:34077942 | *RALY* | A | 0.038 | Liu et al., 2015; |
| 106 | rs4911414 | 20:34141638 | *ASIP* | T | 0.327 | Zhang et al., 2013; Liu et al., 2015 |
| 107 | rs1015362 | 20:34150806 | *ASIP* | T | 0.306 | Sulem et al., 2008; Nan et al., 2009; Pośpiech et al., 2014 |
| 108 | rs6119471 | 20:34197406 | *ASIP* | G | 0.001 | Hart et al., 2013 |
| 109 | rs2424984 | 20:34262569 | *ASIP* | C | 0.143 | Valenzuela et al.; 2010 |
| 110 | rs6058017 | 20:34269192 | *ASIP* | G | 0.143 | Stokowski et al., 2007; Nan et al., 2009; Valenzuela et al., 2010; Pośpiech et al., 2014 |
| 111 | rs2378249 | 20:34630286 | *ASIP* | G | 0.138 | Branicki et al., 2011 |
| 112 | rs4911442 | 20:34767243 | *NCOA6* | G | 0.085 | Eriksson et al., 2010; Hernando et al., 2018 |
| 113 | rs1885120 | 20:34989186 | *MYH7B* | C | 0.038 | Liu et al., 2015; Walsh et al., 2017 |
| 114 | rs7277820 | 21:37208008 | *DSCR9* | G | 0.479 | Liu et al., 2010 |

**References**

Alonso, S., Izagirre, N., Smith-Zubiaga, I., Gardeazabal, J., Díaz-Ramón, J. L., Díaz-Pérez, J. L., Zelenika, D., Boyano, M. D., Smit, N., & de la Rúa, C. (2008). Complex signatures of selection for the melanogenic loci TYR, TYRP1 and DCT in humans. BMC evolutionary biology, 8, 74. https://doi.org/10.1186/1471-2148-8-74

Andersen, J. D., Pietroni, C., Johansen, P., Andersen, M. M., Pereira, V., Børsting, C., & Morling, N. (2016). Importance of nonsynonymous OCA2 variants in human eye color prediction. Molecular genetics & genomic medicine, 4(4), 420–430. https://doi.org/10.1002/mgg3.213

Beleza, S., Johnson, N. A., Candille, S. I., Absher, D. M., Coram, M. A., Lopes, J., Campos, J., Araújo, I. I., Anderson, T. M., Vilhjálmsson, B. J., Nordborg, M., Correia E Silva, A., Shriver, M. D., Rocha, J., Barsh, G. S., & Tang, H. (2013). Genetic architecture of skin and eye color in an African-European admixed population. PLoS genetics, 9(3), e1003372. https://doi.org/10.1371/journal.pgen.1003372

Branicki, W., Brudnik, U., Draus-Barini, J., Kupiec, T., & Wojas-Pelc, A. (2008). Association of the SLC45A2 gene with physiological human hair colour variation. Journal of human genetics, 53(11-12), 966–971. https://doi.org/10.1007/s10038-008-0338-3

Branicki, W., Liu, F., van Duijn, K., Draus-Barini, J., Pośpiech, E., Walsh, S., Kupiec, T., Wojas-Pelc, A., & Kayser, M. (2011). Model-based prediction of human hair color using DNA variants. Human genetics, 129(4), 443–454. https://doi.org/10.1007/s00439-010-0939-8

Duffy, D. L., Iles, M. M., Glass, D., Zhu, G., Barrett, J. H., Höiom, V., Zhao, Z. Z., Sturm, R. A., Soranzo, N., Hammond, C., Kvaskoff, M., Whiteman, D. C., Mangino, M., Hansson, J., Newton-Bishop, J. A., GenoMEL, Bataille, V., Hayward, N. K., Martin, N. G., Bishop, D. T., … Montgomery, G. W. (2010). IRF4 variants have age-specific effects on nevus count and predispose to melanoma. American journal of human genetics, 87(1), 6–16. https://doi.org/10.1016/j.ajhg.2010.05.017

Duffy, D. L., Montgomery, G. W., Chen, W., Zhao, Z. Z., Le, L., James, M. R., Hayward, N. K., Martin, N. G., & Sturm, R. A. (2007). A three-single-nucleotide polymorphism haplotype in intron 1 of OCA2 explains most human eye-color variation. American journal of human genetics, 80(2), 241–252. https://doi.org/10.1086/510885

Edwards, M., Bigham, A., Tan, J., Li, S., Gozdzik, A., Ross, K., Jin, L., & Parra, E. J. (2010). Association of the OCA2 polymorphism His615Arg with melanin content in east Asian populations: further evidence of convergent evolution of skin pigmentation. PLoS genetics, 6(3), e1000867. https://doi.org/10.1371/journal.pgen.1000867

Eiberg, H., Troelsen, J., Nielsen, M., Mikkelsen, A., Mengel-From, J., Kjaer, K. W., & Hansen, L. (2008). Blue eye color in humans may be caused by a perfectly associated founder mutation in a regulatory element located within the HERC2 gene inhibiting OCA2 expression. Human genetics, 123(2), 177–187. https://doi.org/10.1007/s00439-007-0460-x

Eriksson, N., Macpherson, J. M., Tung, J. Y., Hon, L. S., Naughton, B., Saxonov, S., Avey, L., Wojcicki, A., Pe'er, I., & Mountain, J. (2010). Web-based, participant-driven studies yield novel genetic associations for common traits. PLoS genetics, 6(6), e1000993. https://doi.org/10.1371/journal.pgen.1000993

Graf, J., Voisey, J., Hughes, I., & van Daal, A. (2007). Promoter polymorphisms in the MATP (SLC45A2) gene are associated with normal human skin color variation. Human mutation, 28(7), 710–717. https://doi.org/10.1002/humu.20504

Han, J., Kraft, P., Nan, H., Guo, Q., Chen, C., Qureshi, A., Hankinson, S. E., Hu, F. B., Duffy, D. L., Zhao, Z. Z., Martin, N. G., Montgomery, G. W., Hayward, N. K., Thomas, G., Hoover, R. N., Chanock, S., & Hunter, D. J. (2008). A genome-wide association study identifies novel alleles associated with hair color and skin pigmentation. PLoS genetics, 4(5), e1000074. https://doi.org/10.1371/journal.pgen.1000074

Hart, K. L., Kimura, S. L., Mushailov, V., Budimlija, Z. M., Prinz, M., & Wurmbach, E. (2013). Improved eye- and skin-color prediction based on 8 SNPs. Croatian medical journal, 54(3), 248–256. https://doi.org/10.3325/cmj.2013.54.248

Hernando, B., Ibañez, M. V., Deserio-Cuesta, J. A., Soria-Navarro, R., Vilar-Sastre, I., & Martinez-Cadenas, C. (2018). Genetic determinants of freckle occurrence in the Spanish population: Towards ephelides prediction from human DNA samples. Forensic science international. Genetics, 33, 38–47. https://doi.org/10.1016/j.fsigen.2017.11.013

Hysi, P. G., Valdes, A. M., Liu, F., Furlotte, N. A., Evans, D. M., Bataille, V., Visconti, A., Hemani, G., McMahon, G., Ring, S. M., Smith, G. D., Duffy, D. L., Zhu, G., Gordon, S. D., Medland, S. E., Lin, B. D., Willemsen, G., Jan Hottenga, J., Vuckovic, D., Girotto, G., … Spector, T. D. (2018). Genome-wide association meta-analysis of individuals of European ancestry identifies new loci explaining a substantial fraction of hair color variation and heritability. Nature genetics, 50(5), 652–656. https://doi.org/10.1038/s41588-018-0100-5

Ibarrola-Villava, M., Fernandez, L. P., Pita, G., Bravo, J., Floristan, U., Sendagorta, E., Feito, M., Avilés, J. A., Martin-Gonzalez, M., Lázaro, P., Benítez, J., & Ribas, G. (2010). Genetic analysis of three important genes in pigmentation and melanoma susceptibility: CDKN2A, MC1R and HERC2/OCA2. Experimental dermatology, 19(9), 836–844. https://doi.org/10.1111/j.1600-0625.2010.01115.x

Jacobs, L. C., Hamer, M. A., Gunn, D. A., Deelen, J., Lall, J. S., van Heemst, D., Uh, H. W., Hofman, A., Uitterlinden, A. G., Griffiths, C., Beekman, M., Slagboom, P. E., Kayser, M., Liu, F., & Nijsten, T. (2015). A Genome-Wide Association Study Identifies the Skin Color Genes IRF4, MC1R, ASIP, and BNC2 Influencing Facial Pigmented Spots. The Journal of investigative dermatology, 135(7), 1735–1742. https://doi.org/10.1038/jid.2015.62

Jacobs, L. C., Wollstein, A., Lao, O., Hofman, A., Klaver, C. C., Uitterlinden, A. G., Nijsten, T., Kayser, M., & Liu, F. (2013). Comprehensive candidate gene study highlights UGT1A and BNC2 as new genes determining continuous skin color variation in Europeans. Human genetics, 132(2), 147–158. https://doi.org/10.1007/s00439-012-1232-9

Kayser, M., Liu, F., Janssens, A. C., Rivadeneira, F., Lao, O., van Duijn, K., Vermeulen, M., Arp, P., Jhamai, M. M., van Ijcken, W. F., den Dunnen, J. T., Heath, S., Zelenika, D., Despriet, D. D., Klaver, C. C., Vingerling, J. R., de Jong, P. T., Hofman, A., Aulchenko, Y. S., Uitterlinden, A. G., … van Duijn, C. M. (2008). Three genome-wide association studies and a linkage analysis identify HERC2 as a human iris color gene. American journal of human genetics, 82(2), 411–423. https://doi.org/10.1016/j.ajhg.2007.10.003

Kosiniak-Kamysz, A., Pośpiech, E., Wojas-Pelc, A., Marcińska, M., & Branicki, W. (2012). Potential association of single nucleotide polymorphisms in pigmentation genes with the development of basal cell carcinoma. The Journal of dermatology, 39(8), 693–698. https://doi.org/10.1111/j.1346-8138.2012.01559.x

Kukla-Bartoszek, M., Pośpiech, E., Woźniak, A., Boroń, M., Karłowska-Pik, J., Teisseyre, P., Zubańska, M., Bronikowska, A., Grzybowski, T., Płoski, R., Spólnicka, M., & Branicki, W. (2019). DNA-based predictive models for the presence of freckles. Forensic science international. Genetics, 42, 252–259. https://doi.org/10.1016/j.fsigen.2019.07.012

Lao, O., de Gruijter, J. M., van Duijn, K., Navarro, A., & Kayser, M. (2007). Signatures of positive selection in genes associated with human skin pigmentation as revealed from analyses of single nucleotide polymorphisms. Annals of human genetics, 71(Pt 3), 354–369. https://doi.org/10.1111/j.1469-1809.2006.00341.x

Larsson, M., Duffy, D. L., Zhu, G., Liu, J. Z., Macgregor, S., McRae, A. F., Wright, M. J., Sturm, R. A., Mackey, D. A., Montgomery, G. W., Martin, N. G., & Medland, S. E. (2011). GWAS findings for human iris patterns: associations with variants in genes that influence normal neuronal pattern development. American journal of human genetics, 89(2), 334–343. https://doi.org/10.1016/j.ajhg.2011.07.011

Law, M. H., Bishop, D. T., Lee, J. E., Brossard, M., Martin, N. G., Moses, E. K., Song, F., Barrett, J. H., Kumar, R., Easton, D. F., Pharoah, P., Swerdlow, A. J., Kypreou, K. P., Taylor, J. C., Harland, M., Randerson-Moor, J., Akslen, L. A., Andresen, P. A., Avril, M. F., Azizi, E., … Iles, M. M. (2015). Genome-wide meta-analysis identifies five new susceptibility loci for cutaneous malignant melanoma. Nature genetics, 47(9), 987–995. https://doi.org/10.1038/ng.3373

Lin, B. D., Mbarek, H., Willemsen, G., Dolan, C. V., Fedko, I. O., Abdellaoui, A., de Geus, E. J., Boomsma, D. I., & Hottenga, J. J. (2015). Heritability and Genome-Wide Association Studies for Hair Color in a Dutch Twin Family Based Sample. Genes, 6(3), 559–576. https://doi.org/10.3390/genes6030559

Liu, F., van Duijn, K., Vingerling, J. R., Hofman, A., Uitterlinden, A. G., Janssens, A. C., & Kayser, M. (2009). Eye color and the prediction of complex phenotypes from genotypes. Current biology : CB, 19(5), R192–R193. https://doi.org/10.1016/j.cub.2009.01.027

Liu, F., Visser, M., Duffy, D. L., Hysi, P. G., Jacobs, L. C., Lao, O., Zhong, K., Walsh, S., Chaitanya, L., Wollstein, A., Zhu, G., Montgomery, G. W., Henders, A. K., Mangino, M., Glass, D., Bataille, V., Sturm, R. A., Rivadeneira, F., Hofman, A., van IJcken, W. F., … Kayser, M. (2015). Genetics of skin color variation in Europeans: genome-wide association studies with functional follow-up. Human genetics, 134(8), 823–835. https://doi.org/10.1007/s00439-015-1559-0

Liu, F., Wollstein, A., Hysi, P. G., Ankra-Badu, G. A., Spector, T. D., Park, D., Zhu, G., Larsson, M., Duffy, D. L., Montgomery, G. W., Mackey, D. A., Walsh, S., Lao, O., Hofman, A., Rivadeneira, F., Vingerling, J. R., Uitterlinden, A. G., Martin, N. G., Hammond, C. J., & Kayser, M. (2010). Digital quantification of human eye color highlights genetic association of three new loci. PLoS genetics, 6(5), e1000934. https://doi.org/10.1371/journal.pgen.1000934

Maroñas, O., Söchtig, J., Ruiz, Y., Phillips, C., Carracedo, Á., & Lareu, M. V. (2015). The genetics of skin, hair, and eye color variation and its relevance to forensic pigmentation predictive tests. Forensic science review, 27(1), 13–40.

Martin, A. R., Lin, M., Granka, J. M., Myrick, J. W., Liu, X., Sockell, A., Atkinson, E. G., Werely, C. J., Möller, M., Sandhu, M. S., Kingsley, D. M., Hoal, E. G., Liu, X., Daly, M. J., Feldman, M. W., Gignoux, C. R., Bustamante, C. D., & Henn, B. M. (2017). An Unexpectedly Complex Architecture for Skin Pigmentation in Africans. Cell, 171(6), 1340–1353.e14. https://doi.org/10.1016/j.cell.2017.11.015

Mengel-From, J., Børsting, C., Sanchez, J. J., Eiberg, H., & Morling, N. (2010). Human eye colour and HERC2, OCA2 and MATP. Forensic science international. Genetics, 4(5), 323–328. https://doi.org/10.1016/j.fsigen.2009.12.004

Mengel-From, J., Wong, T. H., Morling, N., Rees, J. L., & Jackson, I. J. (2009). Genetic determinants of hair and eye colours in the Scottish and Danish populations. BMC genetics, 10, 88. https://doi.org/10.1186/1471-2156-10-88

Morgan, M. D., Pairo-Castineira, E., Rawlik, K., Canela-Xandri, O., Rees, J., Sims, D., Tenesa, A., & Jackson, I. J. (2018). Genome-wide study of hair colour in UK Biobank explains most of the SNP heritability. Nature communications, 9(1), 5271. https://doi.org/10.1038/s41467-018-07691-z

Nan, H., Kraft, P., Qureshi, A. A., Guo, Q., Chen, C., Hankinson, S. E., Hu, F. B., Thomas, G., Hoover, R. N., Chanock, S., Hunter, D. J., & Han, J. (2009). Genome-wide association study of tanning phenotype in a population of European ancestry. The Journal of investigative dermatology, 129(9), 2250–2257. https://doi.org/10.1038/jid.2009.62

Pośpiech, E., Draus-Barini, J., Kupiec, T., Wojas-Pelc, A., & Branicki, W. (2011). Gene-gene interactions contribute to eye colour variation in humans. Journal of human genetics, 56(6), 447–455. https://doi.org/10.1038/jhg.2011.38

Pośpiech, E., Wojas-Pelc, A., Walsh, S., Liu, F., Maeda, H., Ishikawa, T., Skowron, M., Kayser, M., & Branicki, W. (2014). The common occurrence of epistasis in the determination of human pigmentation and its impact on DNA-based pigmentation phenotype prediction. Forensic science international. Genetics, 11, 64–72. https://doi.org/10.1016/j.fsigen.2014.01.012

Praetorius, C., Grill, C., Stacey, S. N., Metcalf, A. M., Gorkin, D. U., Robinson, K. C., Van Otterloo, E., Kim, R. S., Bergsteinsdottir, K., Ogmundsdottir, M. H., Magnusdottir, E., Mishra, P. J., Davis, S. R., Guo, T., Zaidi, M. R., Helgason, A. S., Sigurdsson, M. I., Meltzer, P. S., Merlino, G., Petit, V., … Steingrimsson, E. (2013). A polymorphism in IRF4 affects human pigmentation through a tyrosinase-dependent MITF/TFAP2A pathway. Cell, 155(5), 1022–1033. https://doi.org/10.1016/j.cell.2013.10.022

Rawofi, L., Edwards, M., Krithika, S., Le, P., Cha, D., Yang, Z., Ma, Y., Wang, J., Su, B., Jin, L., Norton, H. L., & Parra, E. J. (2017). Genome-wide association study of pigmentary traits (skin and iris color) in individuals of East Asian ancestry. PeerJ, 5, e3951. https://doi.org/10.7717/peerj.3951

Ruiz, Y., Phillips, C., Gomez-Tato, A., Alvarez-Dios, J., Casares de Cal, M., Cruz, R., Maroñas, O., Söchtig, J., Fondevila, M., Rodriguez-Cid, M. J., Carracedo, A., & Lareu, M. V. (2013). Further development of forensic eye color predictive tests. Forensic science international. Genetics, 7(1), 28–40. https://doi.org/10.1016/j.fsigen.2012.05.009

Söchtig, J., Phillips, C., Maroñas, O., Gómez-Tato, A., Cruz, R., Alvarez-Dios, J., de Cal, M. Á., Ruiz, Y., Reich, K., Fondevila, M., Carracedo, Á., & Lareu, M. V. (2015). Exploration of SNP variants affecting hair colour prediction in Europeans. International journal of legal medicine, 129(5), 963–975. https://doi.org/10.1007/s00414-015-1226-y

Stokowski, R. P., Pant, P. V., Dadd, T., Fereday, A., Hinds, D. A., Jarman, C., Filsell, W., Ginger, R. S., Green, M. R., van der Ouderaa, F. J., & Cox, D. R. (2007). A genomewide association study of skin pigmentation in a South Asian population. American journal of human genetics, 81(6), 1119–1132. https://doi.org/10.1086/522235

Sturm, R. A., Duffy, D. L., Zhao, Z. Z., Leite, F. P., Stark, M. S., Hayward, N. K., Martin, N. G., & Montgomery, G. W. (2008). A single SNP in an evolutionary conserved region within intron 86 of the HERC2 gene determines human blue-brown eye color. American journal of human genetics, 82(2), 424–431. https://doi.org/10.1016/j.ajhg.2007.11.005

Sulem, P., Gudbjartsson, D. F., Stacey, S. N., Helgason, A., Rafnar, T., Magnusson, K. P., Manolescu, A., Karason, A., Palsson, A., Thorleifsson, G., Jakobsdottir, M., Steinberg, S., Pálsson, S., Jonasson, F., Sigurgeirsson, B., Thorisdottir, K., Ragnarsson, R., Benediktsdottir, K. R., Aben, K. K., Kiemeney, L. A., … Stefansson, K. (2007). Genetic determinants of hair, eye and skin pigmentation in Europeans. Nature genetics, 39(12), 1443–1452. https://doi.org/10.1038/ng.2007.13

Valenzuela, R. K., Henderson, M. S., Walsh, M. H., Garrison, N. A., Kelch, J. T., Cohen-Barak, O., Erickson, D. T., John Meaney, F., Bruce Walsh, J., Cheng, K. C., Ito, S., Wakamatsu, K., Frudakis, T., Thomas, M., & Brilliant, M. H. (2010). Predicting phenotype from genotype: normal pigmentation. Journal of forensic sciences, 55(2), 315–322. https://doi.org/10.1111/j.1556-4029.2009.01317.x

Visser, M., Palstra, R. J., & Kayser, M. (2014). Human skin color is influenced by an intergenic DNA polymorphism regulating transcription of the nearby BNC2 pigmentation gene. Human molecular genetics, 23(21), 5750–5762. https://doi.org/10.1093/hmg/ddu289

Visser, M., Palstra, R. J., & Kayser, M. (2015). Allele-specific transcriptional regulation of IRF4 in melanocytes is mediated by chromatin looping of the intronic rs12203592 enhancer to the IRF4 promoter. Human molecular genetics, 24(9), 2649–2661. https://doi.org/10.1093/hmg/ddv029

Walsh, S., Chaitanya, L., Breslin, K., Muralidharan, C., Bronikowska, A., Pospiech, E., Koller, J., Kovatsi, L., Wollstein, A., Branicki, W., Liu, F., & Kayser, M. (2017). Global skin colour prediction from DNA. Human genetics, 136(7), 847–863. https://doi.org/10.1007/s00439-017-1808-5

Zhang, M., Song, F., Liang, L., Nan, H., Zhang, J., Liu, H., Wang, L. E., Wei, Q., Lee, J. E., Amos, C. I., Kraft, P., Qureshi, A. A., & Han, J. (2013). Genome-wide association studies identify several new loci associated with pigmentation traits and skin cancer risk in European Americans. Human molecular genetics, 22(14), 2948–2959. https://doi.org/10.1093/hmg/ddt142
